# First comprehensive quantification of annual land use/cover from 1990 to 2020 across mainland Vietnam

**DOI:** 10.1038/s41598-021-89034-5

**Published:** 2021-05-11

**Authors:** Duong Cao Phan, Ta Hoang Trung, Van Thinh Truong, Taiga Sasagawa, Thuy Phuong Thi Vu, Dieu Tien Bui, Masato Hayashi, Takeo Tadono, Kenlo Nishida Nasahara

**Affiliations:** 1 Graduate School of Science and Technology, University of Tsukuba, Tennoudai 1-1-1, Tsukuba, Ibaraki 305-8572 Japan; 2 Hydraulic Construction Institute, Vietnam Academy for Water Resources, No. 3, Alley 95, Chua Boc Street, Dong Da district, Hanoi, 116765 Vietnam; 3 Department of Survey, Mapping and Geographic Information, Ministry of Natural Resources and Environment, 2 Dang Thuy Tram Street, Hanoi, 100000 Vietnam; 4 VNU Center for Development in Hoa Lac, Vietnam National University, Hanoi, Thach Hoa Commune, Thach That District, Hanoi, 155500 Vietnam; 5 Forest Inventory and Planning Institute (FIPI), Ministry of Agriculture and Rural Development (MARD), Vinh Quynh, Thanh Tri, Hanoi, 100000 Vietnam; 6 GIS Group, Department of Business and IT, University of South-Eastern Norway, Gullbringvegen 36, N-3800 Bø i Telemark, Norway; 7 Earth Observation Research Center, Japan Aerospace Exploration Agency (JAXA), 2-1-1 Sengen, Tsukuba, Ibaraki 305-8505 Japan; 8 Faculty of Life and Environmental Sciences, University of Tsukuba, Tennoudai 1-1-1, Tsukuba, Ibaraki 305-8572 Japan

**Keywords:** Carbon cycle, Environmental sciences, Hydrology, Natural hazards, Planetary science, Ecology, Climate sciences

## Abstract

Extensive studies have highlighted a need for frequently consistent land cover information for interdisciplinary studies. This paper proposes a comprehensive framework for the automatic production of the first Vietnam-wide annual land use/land cover (LULC) data sets (VLUCDs) from 1990 to 2020, using available remotely sensed and inventory data. Classification accuracies ranged from 85.7 ± 1.3 to 92.0 ± 1.2% with the primary dominant LULC and 77.6 ± 1.2% to 84.7 ± 1.1% with the secondary dominant LULC. This confirmed the potential of the proposed framework for systematically long-term monitoring LULC in Vietnam. Results reveal that despite slight recoveries in 2000 and 2010, the net loss of forests (19,940 km^2^) mainly transformed to croplands over 30 years. Meanwhile, productive croplands were converted to urban areas, which increased approximately ten times. A threefold increase in aquaculture was a major driver of the wetland loss (1914 km^2^). The spatial–temporal changes varied, but the most dynamic regions were the western north, the southern centre, and the south. These findings can provide evidence-based information on formulating and implementing coherent land management policies. The explicitly spatio-temporal VLUCDs can be benchmarks for global LULC validation, and utilized for a variety of applications in the research of environmental changes towards the Sustainable Development Goals.

## Introduction

Information about land use/land cover (LULC) and its dynamic changes are fundamental to a variety of studies on environmental issues^1^ such as climate change^2^, drought and flood^3^, and carbon emissions^4^. That is, frequently updated accurate LULC products provide policymakers with a profound understanding of the complex interplay between land use/cover change (LUCC) and its risk, which helps to inform coherent policies for the sustainable management of land resources^5–7^.

The ready availability of remote sensing data and computing technologies opens a great era in cost-effective mapping LULC at a broad scale. Numerous algorithms have been developed to improve LULC classification, e.g. Spatial Temporal Adaptive Algorithm^8^, Automatic Land Cover Classification Method^9^, and Apply Change-vector Analysis in Posterior Probability Space^10^. Together with the development of these complex algorithms, special projects have been designed for large-scale land cover assessment. For example, at the 10-m spatial resolution, several attempts have been made to publish 13-category LULC maps of Europe^11,12^ and global LULC maps^13^ using a great set of Sentinel MSI images. For a coarser spatial resolution (30 m), there are quality multi-category LULC products, including the National Databases of the United States^14–17^, and the GlobeLand30 global product of 10-category LULC^18^. Nonetheless, owing to the computational restriction and the limitations of representative reference data to train and test classifiers, these products have not reflected consistently and frequently the detailed patterns and characteristics of LULC at local or national scales^19,20^. Also, due to the predefined research periods and differences in the land cover classification systems (LCCSs), these products seldom meet the prime requirement of projects’ specific objectives.

Recently, a remarkable performance in cloud computing has advanced LULC observation sciences. For example, the National Aeronautics and Space Administration Earth Exchange (NASA-NEX) and Amazon Web Service (AWS) allow analysts to access and process the NASA Earth Observation (EO) data on the cloud^21^. More importantly, Google Earth Engine (GEE) provides an outstanding cloud computing platform with open access to a variety of EO data. Thanks to the potential of big data processing of these platforms, researchers have completed extensive studies at greater extent, for example, on urban change monitoring^22^, cultivated land mapping^23^, and forest disturbance detection^24^. Multi-category land cover products were also produced such as a 13-category land cover map of South-east Asia covering 11 nations^25^. Although the overall accuracy of such products reaches up to 86%, the authors identify limitations regarding the insufficiency of high-quality reference data for time-series analyses^26^. Therefore, very few studies have conducted for multi-temporal LULC mapping at a broad scale.

The quantity and quality of training data play an essential role in the production of LULC maps. Yet, collecting sufficient and precise training data requires considerable efforts, especially at large scales and multiple periods^27^. Several attempts have been proposed methods that allow to collect cost-effectively high-quality training data. Bagan et al. (2019) extracted training data from a previous land cover map and utilized them for mapping new LULC products^28^. While the authors applied a bi-temporal spectral measurement to decrease the bias of extracted training data, the accuracy of these data may not be ensured due to the inherent classification errors of the previous maps^29^. To enhance the effectiveness of training data collection, Huang et al. (2020) used spectral similarity and distance indicators to detect the changed and unchanged training sites, and thus kept the unchanged ones as migrated training data^27^. The measurement was applied for the availability of Landsat TM images. Results showed that the accuracies of the migrated training data obtained over 92.98% and the classification map which used the migrated training data had a similar overall accuracy of 71.42% to that used ground-truth data in 2010. Nevertheless, these results were validated by outdated maps, namely the ESA global CCI land cover data sets, which may contain inherent classification errors. In addition, using the Sentinel MSI images, Ghorbanian et al. (2020) employed the same approach to migrate Iran-wide training data from 2017 to 2019^26^. The classified map that utilized the migrated training data obtained a great accuracy of 91.35%. Despite the potential of the automatic training migration method, it is still not known whether this method can be applied for multi-sensor data sources such as the different Landsat sensors or the harmonized Landsat and Sentinel images^27^. Therefore, an exploration of the potential training migration method for multi-sensor remote sensing data is integral for a time-series assessment of multi-category LULC dynamics at a large scale.

Given the ideas, this research aim is to explore the potential training migration method for multi-sensor remote sensing data and then produce the first Vietnam-wide annual land use/cover data sets (VLUCDs) from 1990 to 2020 as a case study. In Vietnam, remotely sensed data have been utilized to produced quality LULC products, but most products cover a small area of the country or a few predefined periods^30–32^. The previous inter-provincial LULC data sets were seven-category LULC maps for the central and southern Vietnam in 2007 and 2017, and the northern Vietnam in 2007 and 2015^20^. More recently, Vietnam-wide maps were produced to map annual forest cover from 2015 to 2019^19,33^. Despite the potential of these products, due to the primary focus on forest monitoring, the classification accuracy of non-forest LULC categories may be insufficient for other applications. Meanwhile, there has been a highly dynamic LUCC which varies among different regions in Vietnam. Despite the report of continuous net forest gain by the Ministry of Agricultural and Rural Development (MARD)^34^, a systematically comprehensive review has reported forest loss in Vietnam^35,36^. We do not know exactly the rates and patterns of changes at the nation scale. Hence, timely, accurate, and comprehensive LULC products can provide a profound understanding of LUCC patterns and processes. This information can supports policymakers in forming crucial decisions on sustainable development and resource management. The maps may be benchmarks for quantifying regional and global land cover products.

The central novelty of this paper is to propose a new framework for the automatic nationwide annual LULC monitoring and provide the results of the first VLUCDs and LUCC over the recent three decades. There are major tasks: (1) Data preparation; (2) Design a proper LCCS and reference data; (2) Proposing a consistent framework for the automatic production of the VLUCDs; (3) Creating and validating the VLUCDs, and; (4) Detecting profound changes in LULC since 1990. We developed a new random-forest-based classification approach to classify the wide availability of Landsat Thematic Mapper (TM), Enhanced Thematic Mapper Plus (ETM+) and Operational Land Imager (OLI), and Sentinel C-band Synthetic Aperture Radar Ground Range Detected (SAR GRD) and MultiSpectral Instrument (MSI) time-series images over the study period. We describe fully our implementation in the method section.

## Results

### The accuracy of the first VLUCDs

**Figure 1:**
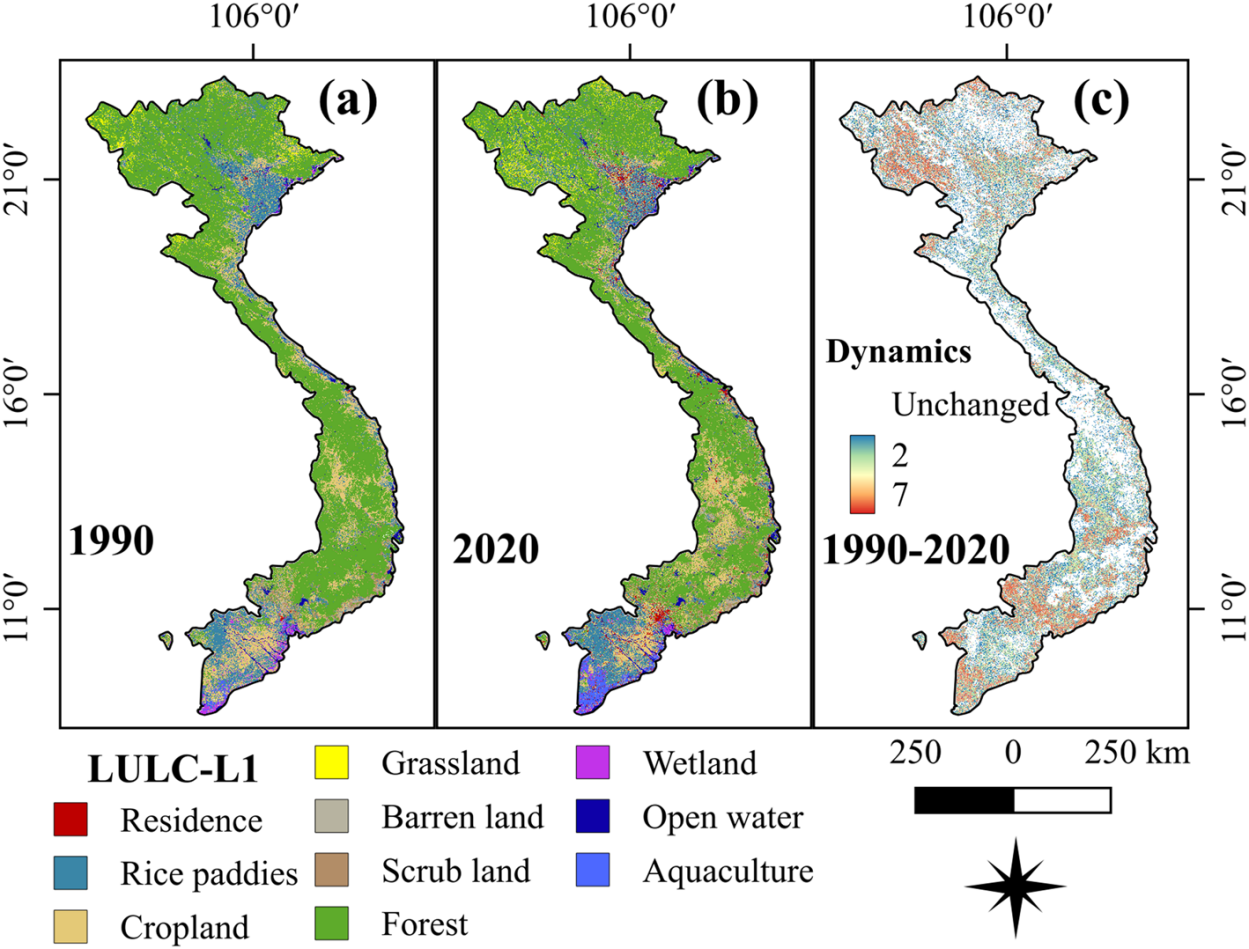
(**a**, **b**) show the level-1 Vietnam-wide LULC maps in 1990 and 2020 produced from a fusion of Landsat TM, ETM + and OLI, and Sentinel SAR GRD and MSI images with the random-forest-based algorithm. (**c**) presents a spatial–temporal dynamic change in LULC from 1990 to 2020 in Vietnam. This figure is generated using QGIS 3.18.0-Zurich (https://qgis.org/en/site/) while the country boundary is extracted from the GADM (https://gadm.org/about.html).

**Figure 2:**
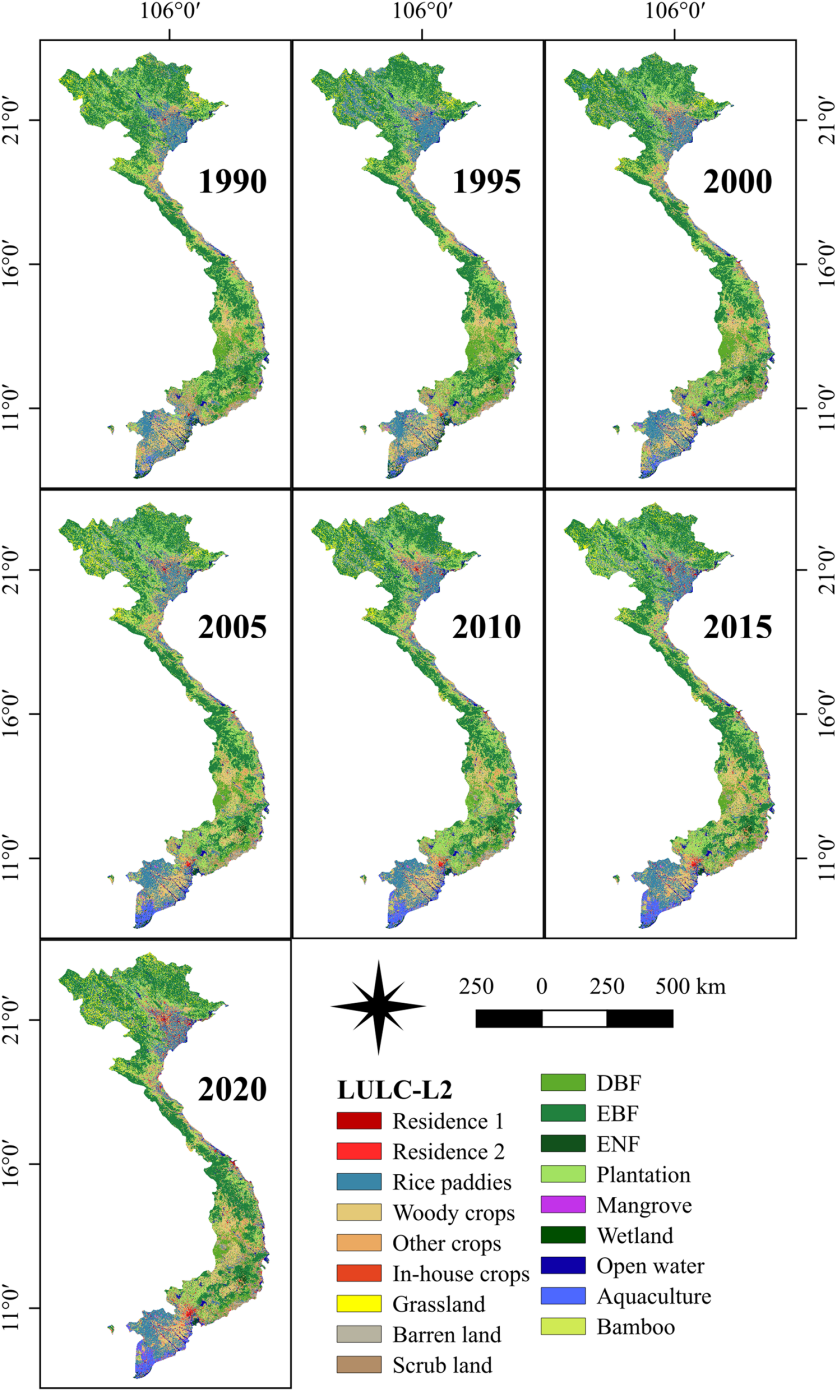
The level-2 Vietnam-wide LULC maps in 1990, 1995, 2000, 2005, 2010, 2015 and 2020 produced from a fusion of Landsat TM, ETM + and OLI, and Sentinel SAR GRD and MSI images with the random-forest-based algorithm. This figure is generated using QGIS 3.18.0-Zurich (https://qgis.org/en/site/) while the country boundary is extracted from the GADM (https://gadm.org/about.html).

Utilizing ground-based data and all the freely available remotely sensed images, we have provided a coherent method and the results of the first VLUCDs. The proposed method generated consistently spatio-temporal LULC maps, using a definitive LCCS designed with reference to end-users’ recommendations and a standard LCCS^37^. For a visual presentation, level-1 VLUCDs of the year 1990 and 2020 are presented in Fig. 1. The 5-year-interval maps (1990, 1995, 2000, 2005, 2010, 2015, and 2020) of level-2 VLUCDs are presented in Fig. 2. The level-1 and level-2 VLUCDs included ten categories of primary dominant land use/cover (PDLC) and eighteen categories of secondary dominant land use/cover (SDLC), respectively (see detail in Supplementary Table S1).

**Figure 3:**
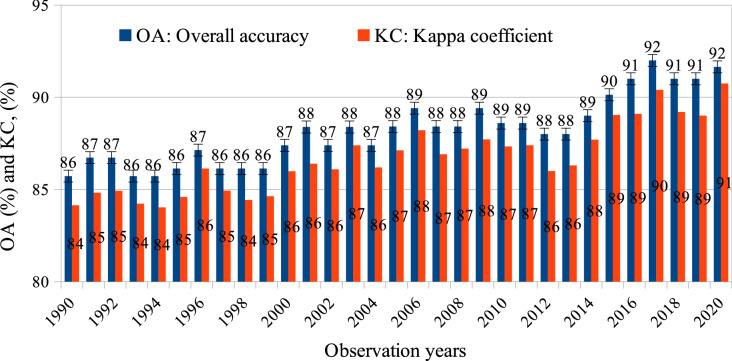
The overall accuracy (OA) and kappa coefficient (KC) of the level-1 Vietnam-wide annual LULC maps produced from the all freely available Landsat TM, ETM + and OLI, and Sentinel SAR GRD and MSI images with the random-forest-based algorithm. The OA and KC are obtained by using a confusion matrix and a stratified validation method with independent samples (1050 points/LULC category). The bars indicate uncertainties of OA measured with a 95% confidence interval.

**Figure 4:**
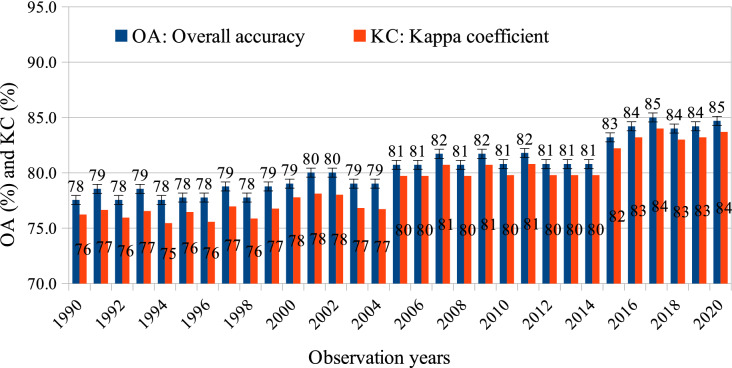
The overall accuracy (OA) and kappa coefficient (KC) of the level-2 Vietnam-wide annual land use/cover maps produced from all the freely available Landsat TM, ETM + and OLI, and Sentinel SAR GRD and MSI images with the random-forest-based algorithm. The OA and KC are obtained by using a confusion matrix and a stratified validation method with independent samples (1050 points/LULC category). The bars indicate uncertainties of OA measured with a 95% confidence interval.

The reliability of the VLUCDs was evaluated by using both visual interpretations and statistical approaches. Based on high-resolution satellite images in Google Earth, we found that the VLUCDs were clear and noise-free. A confusion matrix method with stratified random sampling (1050 points/LULC category) was utilized to independently validate classification accuracy. Statistical metrics were measured, namely producer accuracy (PA), user accuracy (UA), F1 score, overall accuracy (OA), standard error (SE), and kappa coefficient (KC). These metrics of the level-1 and level-2 LULC maps of the year 2020 are fully described in Tables 1 and 2. Meanwhile, overall obtained OA, KC, and uncertainty of the level-1 and level-2 VLUCDs are presented briefly in Figs. 3 and 4. Uncertainty of measurement was estimated with a 95% confidence interval. Specifically, the OA of the level-1 and level-2 LULC maps ranged from 77.6 ± 1.2% to 84.7 ± 1.1%, and 85.7 ± 1.3% to 92.0 ± 1.2% over the study period, respectively.

**Table 1:** Confusion matrix of the 2020 Vietnam-wide land use/cover map (Level 1) created from the integration of Landsat OLI, Sentinel SAR GRD and MSI satellite images with the random-forest-based algorithm. PA: Producer accuracy (%); PA: Producer accuracy (%); UA: User accuracy (%); SEM: Standard error of the mean for UA; F1: F1 score; Overall accuracy: 91.6%, and Kappa coefficient: 90.7 (%). RL: Residence; RP: Rice paddies; CL: Cropland; GL: Grassland; BL: Barren land; SL: Scrubland; FL: Forest land; WL: Wetland; OW: Open water; AC: Aquaculture.

		Land cover map									
		RL	RP	CL	GL	BL	SL	FL	WL	OW	AC
Reference data	RL	988	3	21	1	6	4	0	2	0	2
	RP	1	988	26	1	4	1	8	0	0	1
	CL	27	18	772	1	28	38	17	1	0	0
	GL	1	15	57	990	15	8	64	0	0	0
	BL	25	8	14	44	980	11	11	0	0	1
	SL	0	4	23	13	17	986	44	1	0	3
	FL	1	2	14	0	0	2	853	0	0	0
	WL	3	2	119	0	0	0	53	1040	0	44
	OW	0	3	0	0	0	0	0	1	1031	4
	AC	4	7	4	0	0	0	0	5	19	995
	PA	96.2	95.9	85.6	86.1	89.6	90.4	97.8	82.5	99.2	96.2
	UA	94.1	94.1	73.5	94.3	93.3	93.9	81.2	99.0	98.2	94.8
	SEM	0.7	0.7	1.4	0.7	0.8	0.7	1.2	0.3	0.4	0.7
	F1	0.95	0.95	0.79	0.90	0.91	0.92	0.89	0.90	0.99	0.96

**Table 2:** Confusion matrix of the 2020 Vietnam-wide LULC map (Level 2) created from the integration of Landsat OLI, and Sentinel SAR GRD and MSI satellite images with the random-forest-based algorithm. PA: Producer accuracy (%); UA: User accuracy (%); SEM: Standard error of the mean for UA; F1: F1 score; Overall accuracy: 84.7%, and Kappa coefficient: 83.8 (%). R1: Residence 1; R2: Residence 2; RP: Rice paddies; WC: Woody crops; OC: Other crops; IC: In-house crops; GL: Grassland; BL: Barren land; SL: Scrubland; DBF: Deciduous broadleaf forest; EBR: Evergreen broadleaf forest; ENF: Evergreen needleleaf forest; PL: Plantation land; MF: Mangrove forest; IW: Inland wetland; OW: Open water; AC: Aquaculture; BA: Bamboo areas.

		Land cover map								
		R1	R2	RP	WC	OC	IC	GL	BL	SL
**Reference data**	R1	804	137	3	0	10	0	2	6	1
	R2	212	848	4	17	12	3	1	7	8
	RP	0	1	932	5	27	1	3	5	0
	WC	0	2	12	674	45	0	6	3	6
	OC	0	2	27	26	801	2	1	5	11
	IC	15	34	9	15	26	1044	0	26	84
	GL	0	0	14	39	45	0	975	17	9
	BL	16	14	11	8	12	0	40	960	10
	SL	0	1	3	13	8	0	17	16	898
	DBF	1	0	1	21	5	0	0	2	17
	EBF	0	0	1	8	1	0	2	1	1
	ENF	0	0	0	37	0	0	0	0	5
	PL	0	0	12	12	16	0	1	1	0
	MF	0	6	2	7	2	0	0	1	0
	IW	0	3	7	160	35	0	0	0	0
	OW	1	0	2	0	0	0	0	0	0
	AC	1	2	10	0	3	0	0	0	0
	BA	0	0	0	8	2	0	2	0	0
	PA	83.2	76.1	95.0	84.6	89.4	82.9	83.4	88.2	88.4
	UA	76.6	80.8	88.8	64.2	76.3	99.4	92.9	91.4	85.5
	SEM	1.3	1.2	1.0	1.5	1.3	0.2	0.8	0.9	1.1
	F1	0.80	0.78	0.92	0.73	0.82	0.90	0.88	0.90	0.87

		Land cover map								
		DBF	EBF	ENF	PL	MF	IW	OW	AC	BA
**Reference data**	R1	0	0	0	0	0	0	0	3	0
	R2	0	0	0	0	0	2	0	1	0
	RP	2	0	0	5	0	0	0	0	0
	WC	5	7	0	35	2	0	0	0	0
	OC	3	2	0	15	0	1	0	0	0
	IC	6	0	0	0	0	0	0	0	0
	GL	2	24	0	43	0	0	0	0	1
	BL	6	1	0	5	0	0	1	4	0
	SL	33	12	0	13	0	0	0	2	0
	DBF	980	30	4	35	0	0	0	0	0
	EBF	2	355	1	41	0	0	0	0	6
	ENF	9	202	1045	19	0	0	0	0	0
	PL	2	39	0	603	2	3	0	0	0
	MF	0	2	0	17	1036	1	1	3	0
	IW	0	0	0	100	2	1042	0	95	0
	OW	0	0	0	0	1	0	1028	3	0
	AC	0	0	0	0	6	1	20	939	0
	BA	0	376	0	119	1	0	0	0	1043
	PA	89.4	84.7	79.3	87.3	96.1	72.2	99.3	95.6	67.2
	UA	93.3	33.8	99.5	57.4	98.7	99.2	97.9	89.4	99.3
	SEM	0.8	1.5	0.2	1.5	0.4	0.3	0.4	0.9	0.3
	F1	0.91	0.48	0.88	0.69	0.97	0.84	0.99	0.92	0.80

With the numerous LULC types and long-term observation, these results constituted an outstanding achievement^38^. For the detailed LULC products (Level 2), open water and mangrove had the highest levels of accuracy, accounting for over 96% in both the PA and UA. This successful classification may be explained by the benefits of spectral indices (Table 4) such as the NDWI, WRI, and NDPI, which could distinguish open water from land, aquaculture ponds, and others, whereas mangrove can be accurately identified with the MVI^39^. This was followed by rice paddies, which had an accuracy of above 90%. It seems possibly that rice is frequently cultivated in flat terrain, where is not be affected by topographic problems such as the shadows of mountainsides. The spectral reflectance of rice is also stable^40^. Although the model could separate forests from others, it tended to misclassifying different forests. Another limitation is to classify plantation forests from woody crops, which is also found by numerous studies^41,42^. Likewise, the model could not entirely divide the different types of residential areas, but it showed a clear separation of the residential areas from others. To increase the accuracy of the maps for further analyses, we combined these mixed categories. This combination obtained an increase in accuracy of approximately 6% with a few losses of detail in LULC types.

### Distribution and trend of LUCC

Change detection was conducted to comprehend LUCC patterns and processes. To this end, the level-1 (PDLC) Vietnam-wide annual LULC data sets (L1-VLUCDs) was utilized for further analysis in this study. Although the annual maps are integral to obtain the process of LULC dynamic changes in Vietnam, the five-year-interval land cover products in 1990, 1995, 2000, 2005, 2010, 2015, and 2020 were utilized to acquire a more profound change visualization. A post-classification analysis method was employed to measure the spatio-temporal LUCC and the percentage of changes.

**Figure 5:**
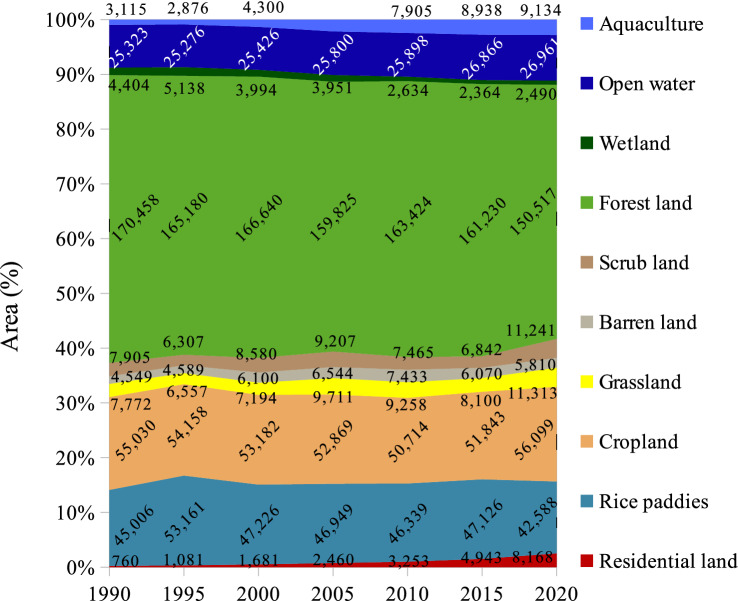
Temporal distribution of LULC across Vietnam extracted from the level-1 Vietnam-wide annual LULC data sets. The data labels represent the area of each LULC category (km^2^) in the year 1990, 1995, 2000, 2010, 2015, and 2020.

The spatial distributions and the patterns of Vietnam LULC are shown in Fig. 1. The temporal distribution of the net changes in LULC from 1990 to 2020 is presented in Fig. 5. The most dominant LULC was forests, accounting for approximately half area of the entire country. This was followed by croplands (16.3%), rice fields (14.2%), and open water (including parts of saltwater, 8.1%). Grassland and scrubland occupied a relatively similar proportion (2.8%) while the smallest LULC was residential areas (1.3%).

What can be clearly seen in Fig. 5 is the steady decline of forest area from 170,458 km^2^ in 1990 to 150,517 km^2^ in 2020. The area of wetlands experienced a slight increase from 4404 km^2^ in 1900 to 5138 km^2^ in 1995, followed by a continual decrease to 2490 km^2^ in 2020. In contrast, there was a sharp increase in the area of aquaculture and residential land, accounting for approximately three and ten times over the three decades. The area of open water showed a slight rise while there was a small fluctuation in the area of the other land types.

**Figure 6:**
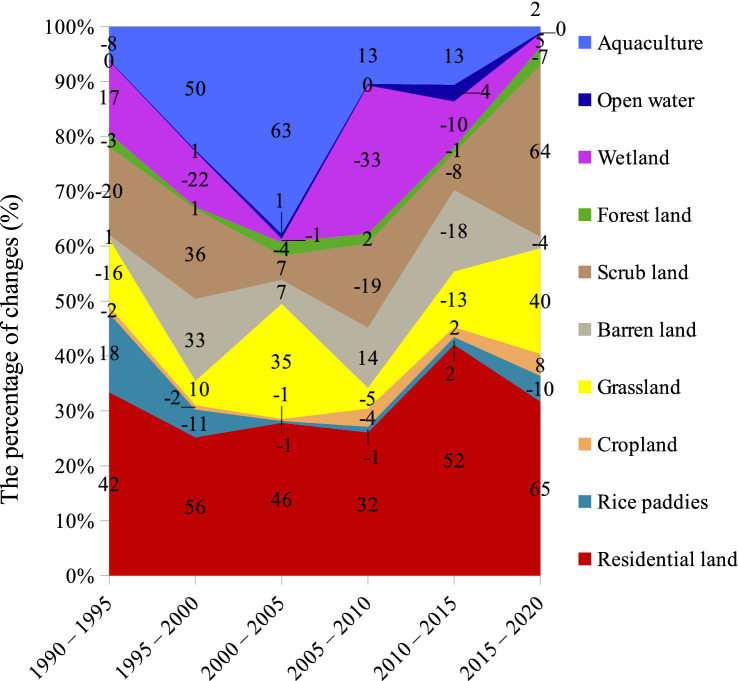
Temporal dynamics of net changes in LULC across Vietnam, extracted from the level-1 Vietnam-wide annual LULC data sets in the years 1990, 1995, 2000, 2010, 2015, and 2020. The data labels represent the percentage of changes (%) within five-year intervals. The positive and negative values indicate an increase and a decrease, respectively.

**Figure 7:**
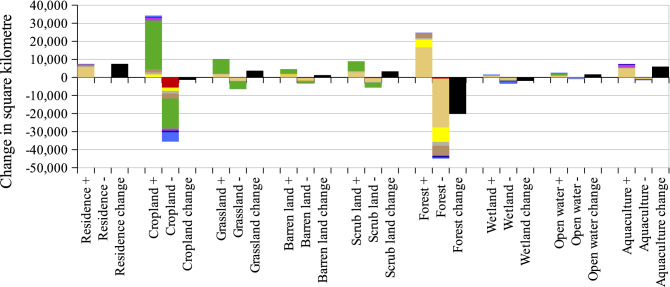
LULC gain/loss and conversions between 1990 and 2020; “ + ” means gain and “-” means loss in area (km^2^).

Meanwhile, Fig. 6 reveals the highly dynamic change in LULC in Vietnam. The most considerable change was the area of urban land, which increased by about 50% over each five-year interval. Interestingly, the graph shows substantially opposite trends between aquaculture and wetlands. The area of aquaculture decreased 8% whereas the wetlands expanded 17% from 1990 to 1995, followed by a 50% increase in aquaculture but a 22% decrease in wetlands by 2000. Forest cover had undergone an up-and-down variation by 2015, but it has presented a remarkable drop until now. It is noticed that the percentage of change in the forest cover was insignificant, but its dynamic areas were remarkable (Fig. 7).

### Major spatio-temporal LULC dynamics

**Figure 8:**
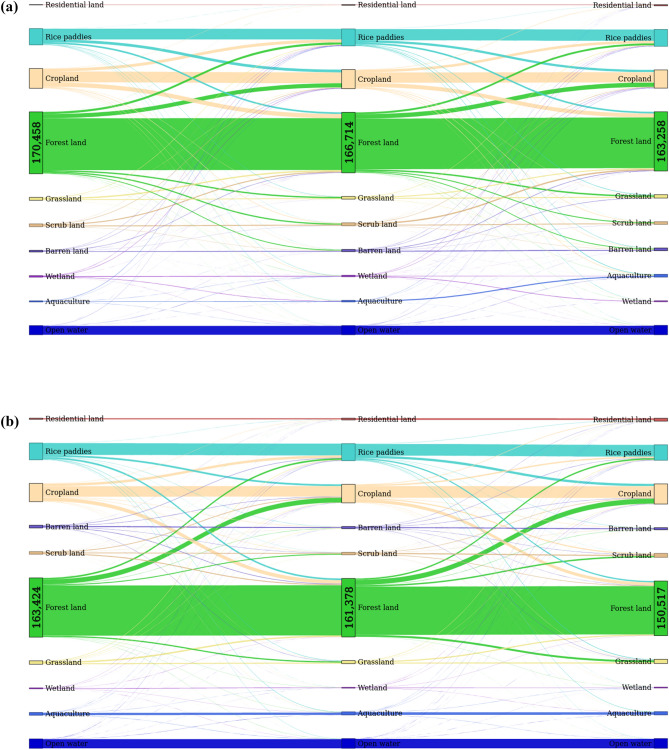
Temporal gross land use/cover conversions in Vietnam. (**a**, **b**) represent transitions among different land types from 1990 to 2010, and from 2010 to 2020, respectively. The numbers indicate the areas of forests (km^2^).

Vietnam’s LULC has experienced a considerable change over the past 30 years. Figure 1c shows the spatio-temporal dynamic changes. The north and south were the most dynamic areas, especially the western north and the most south. The dynamic conversions among different LULC can be seen in Fig. 7. There was a fundamentally dynamic conversion between forests and croplands. Forest areas remarkably reduced while residential and aquaculture land significantly increased. To easily visualize the LULC transformation, we created additional data in Fig. 8. It is noticed that a considerable proportion of forests was converted into croplands while a major driver of wetland loss resulted in the expansion of aquaculture. Residential lands mainly expanded on the areas of rice, croplands, and barren lands, which are located nearby coastlines. To acquire a more detailed visualization of change patterns, few hotspot regions were extracted throughout the country to discuss the change pattern and processes.

## Discussion

Large-scale annual LULC information is integral for understanding the land dynamic process, thus supporting the strategies of land management. In reviewing the literature, little consistent multi-spatio-temporal LULC data was found on a national or regional scale. In this study, a comprehensive framework is developed to produce consistently Vietnam-wide annual LULC data sets, using remote sensing and ground-based data. Results show that surface reflectance images can provide a coherent time-series data set as long as they are atmospherically corrected. Surprisingly, although all available Landsat images of the entire year are utilized, there are data gaps due to cloud and shadow masking areas. These gaps might affect the accuracy of classified maps even though gaps have been filled by ancillary data such as terrain indices. This issue may be explained by the fact that Vietnam is one of the cloudiest countries in the world^43^. However, the harmonious blend of Landsat OLI, Sentinel SAR GRD and MSI has filled such missing-data gaps since 2015, which can also improve the accuracy of mapping (Figs. 3, 4). Besides, the training migration model significantly reduced the cost and efforts in collecting training data.

**Figure 9:**
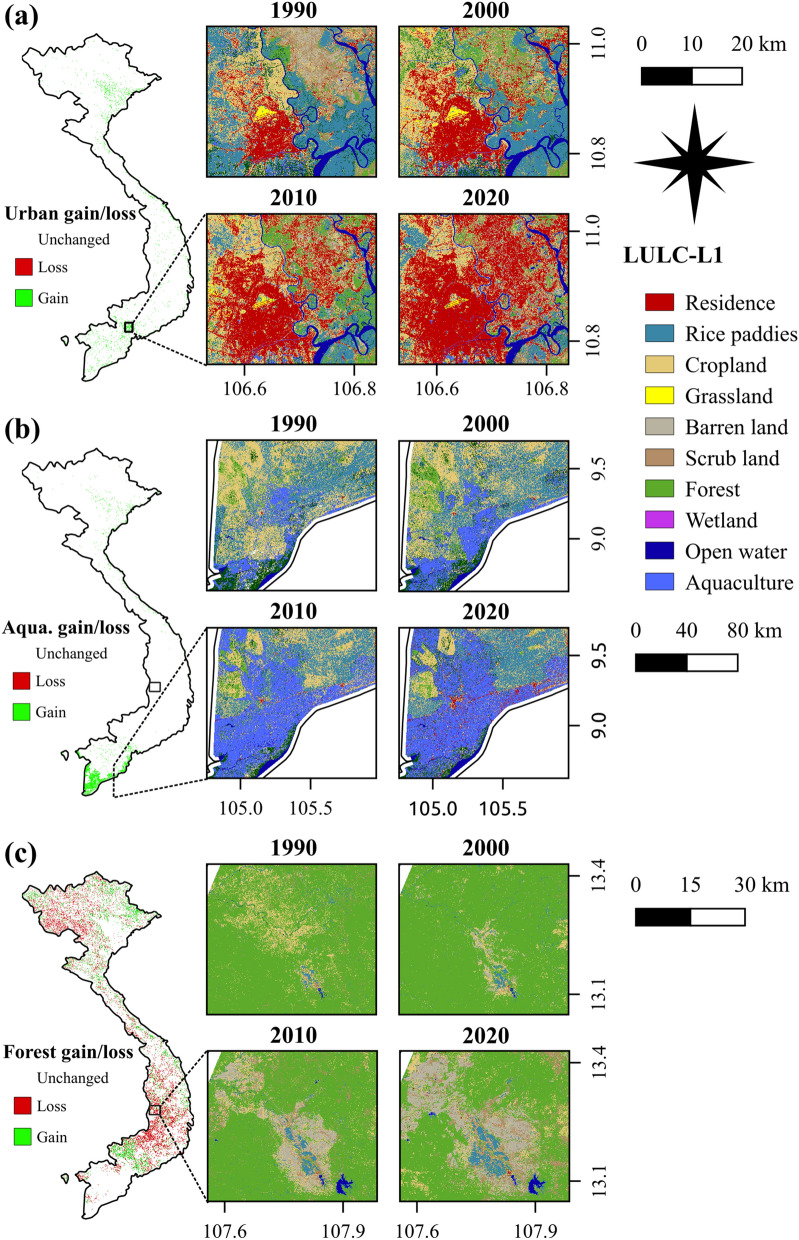
Spatial–temporal dynamics (left) and change pattern (right) of LULC in (**a**) residential land, (**b**) aquaculture land, and (**c**) forests land in Vietnam. This figure is generated using QGIS 3.18.0-Zurich (https://qgis.org/en/site/) while the country boundary is extracted from the GADM (https://gadm.org/about.html).

Regarding change patterns, the rapid development of urbanization is considered an essential interest in Vietnam. The expansion of urban areas has frequently occurred in the capital and regional capitals, namely Hanoi, Hai Phong, Da Nang, Ho Chi Minh (HCM), and Can Tho cities. Herein, we analyse the process of change in HCM as a typical example. As shown in Fig. 9a, the growth of urban land has remarkably increased since the 1990s. This may be explained by the fact that the introduction of new policies known as “Renovation” (1986), which has promoted the development of socio-economic factors, followed by a massive population migration to cities^44^. The urbanization has primarily taken place on croplands, which agrees with the findings of previous studies^30,44–46^. The development is predicted to accelerate over developing regions, which causes the loss of croplands, and thus may threaten sustainability and livelihoods^45^.

Another considerable change is the uncontrolled development of agricultural and aquaculture land, especially in the Vietnamese Mekong Delta (VMD). As part of the third-largest basin in the world, the VMD plays an integral role in the contribution of agricultural products, due to its favourable natural condition of 700 km coastline and a dense network of rivers. In fact, rice and shrimp have contributed to a vital position in Vietnam’s economic development for decades^47,48^. However, the intensification of uncontrolled aquatic farming has caused significant changes in LULC across the region, especially along coastal zones since 2000 (Fig. 9b). There are several possible explanations for this finding. In 2000 and 2001, the Vietnamese government proposed resolutions 09/ND-CP and 1116, which replaced low-value (e.g. rice) to high-value (e.g. shrimp and fruit) agricultural production, encouraging farmers to transform certain coastal areas into aquatic production. Also, due to the high profits of shrimp (200,000 VND/kg) in comparison to traditional crops such as rice (5000 VND/kg)^30^, numerous inland areas were converted into aquatic farming. Local people illegally cut mangrove forests to expand aquaculture in several coastal regions^49^. Since 2010, there was not only an increase in aquaculture, but also a significant conversion of other croplands into rice paddies (Fig. 9b). These changes resulted in the formulation of another policy aiming at the increase of intensive rice and fish farming in 2012^50,51^. These findings indicate that the development of socio-economic policies is considered as the primary reasons driving LUCC. Land policies, therefore, should be formed and implemented in serious consideration of regional socio-economic and environmental development.

Contrary to the expectations, this study indicates a net area loss of forests instead of the constant increase reported by the Vietnamese Ministry of Agriculture and Rural Development (MARD). Despite the net forest regrowth in 2000 and 2010, the forest cover has undergone a decrease in recent years. Also, the area of forests in this study is greater than the data reported by the MARD. These inconsistencies are due to several reasons, especially the difference of forest definitions. The MARD excluded agricultural (e.g., rubber), aqua-cultural ecosystems, scattered trees, bamboos, and palms, etc. from forests^34^. These non-forest lands, covering a relatively large area of the country (e.g., 10 km^2^ of rubber only; 2017), were highly dynamic^19^ but not fully reported by the MARD. In 2008, the revised definition of forests set a minimum of 10% tree cover as forests, instead of 30% tree cover in the previous definition^34^. Since 2016, they started to include certain agricultural lands managed by the Vietnam Administration of Forestry in the forest lands but without forest cover^51^. These revisions likely resulted in an increase in the reporting data of forests. Furthermore, our results show that deforestation occurred in numerous regions. Figure 9c presents a representative example of forest loss in the central highlands. There has been a constant decrease in forest cover due to the expansion of rice paddies, barren lands, and croplands. This finding of forest loss corroborates the discoveries of a great deal of previous work in LULC observation covering Vietnam^52,53^.

Regarding limitations of this study, we could not estimate the benefit of the individual sensor’s characteristics although the harmonious blend of the Landsat OLI, Sentinel SAR GRD and MSI images can fill data gaps and improve the accuracy of mapping classification. Moreover, instead of using ground-truth data, we validated the annual maps of the year 1990 to 2014 with the data collected by visual interpretation. Although great and careful efforts were applied in the collection procedure, errors might not be inevitable due to the restricted high-resolution images in Google Earth, especially before 2000. In addition, we have utilized the random forest algorithm to eliminate the less important input features, but there are relatively numerous remaining features, resulting in a high computational cost. For future work, the nationwide annual multi-category LULC maps and overall change detection were successfully developed, but further research should be undertaken to investigate the drivers of LULC changes in more detail for individual land cover. Finally, deep learning neural networks are expected to be applied for large-scale LULC mapping.

## Conclusion

Regularly updated and accurate LULC information is fundamental to interdisciplinary studies. The recent advancement of remote sensing and computational science has improved the mapping capacity of LULC. This study set out to develop a new framework for automatically monitoring nationwide annual LULC and provide the first VLUCDs over the past 30 years. To this end, we utilized ground-based data, the informative Landsat TM, ETM + and OLI, and Sentinel SAR GRD and MSI images, after comprehensively assembled and preprocessed on the GEE platform. Then, we developed a new random-forest-based method and an automatic training migration model (ATMM) to map Vietnam LULC. The obtained VLUCDs had overall accuracies ranging from 85.7 ± 1.3% to 92.0 ± 1.2% with the ten primary dominant land use/cover and 77.6 ± 1.2% to 84.7 ± 1.1% with the eighteen secondary dominant land use/cover. This confirms the potential of the proposed framework for systematically long-term monitoring of LULC in Vietnam. Results reveal that there was a decrease in the area of forests (19,940 km^2^) and wetlands (1914 km^2^) whereas the area of aquaculture and urban increased approximately three and ten times over the three decades, respectively. The deforestation was mainly due to the expansion of croplands, which were in return replaced by numerous built-up areas. The rapid growth of aquaculture was considered as a main driver of wetland loss. The explicit spatio-temporal benchmark of the VLUCDs can be utilized for a tremendous variety of applications in the research of environmental changes towards the Sustainable Development Goals. In addition, the ATMM allows analysts to remarkably save time, cost, and labour for collecting sufficient and representative training data. This proposed method is possible to apply for a multi-temporal LULC assessment at a broader scale.

## Materials and methods

**Figure 10:**
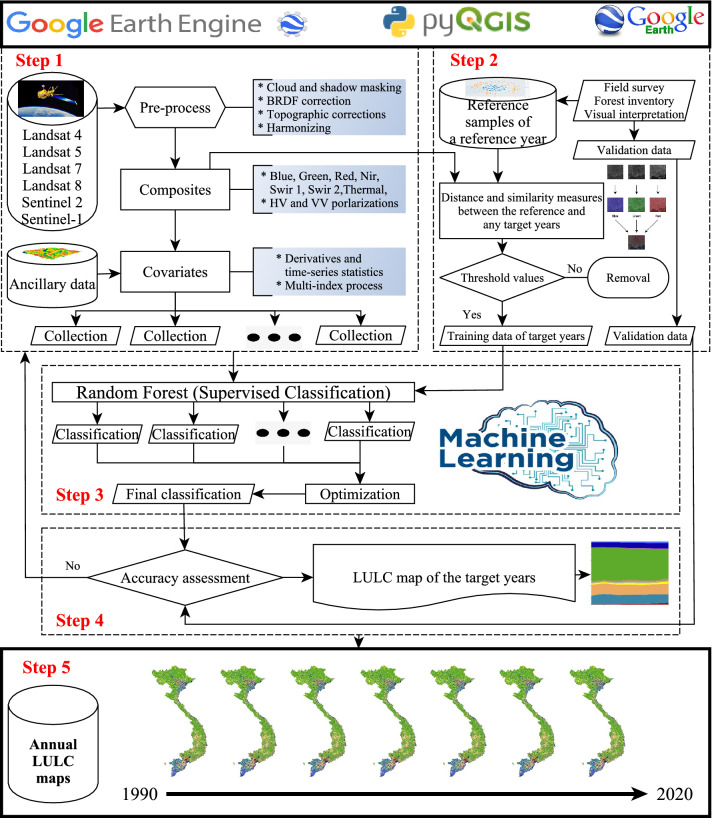
The overall workflow for automatic Vietnam-wide annual land use/cover mapping and monitoring, using Landsat TM, ETM + and OLI, and Sentinel SAR GRD and MSI images with the random-forest-based algorithm. This figure is generated using yEd Graph Editor (https://www.yworks.com/products/yed). The logos of the Google Earth Engine, pyQGIS, Google Earth, and Machine Learning is taken from https://earthengine.google.com/, https://automating-gis-processes.github.io/site/develop/lessons/L7/overview.html, https://logos.fandom.com/wiki/Google_Earth, and https://www.pngitem.com/middle/hRJJRRJ_machine-learning-course-near-me-machine-learning-logo/, respectively.

The overall method is presented in Fig. 10 with major steps: (1) Data preparation; (2) Defining a proper LULC classification system and reference data; (3) Proposing a consistent framework for the automatic production of Vietnam-wide annual LULC data sets from 1990 to 2020; (4) Creating and validating the VLUCDs, and; (5) Generating major change pattern and processes of LULC over the past three decades.

### Study area

**Figure 11:**
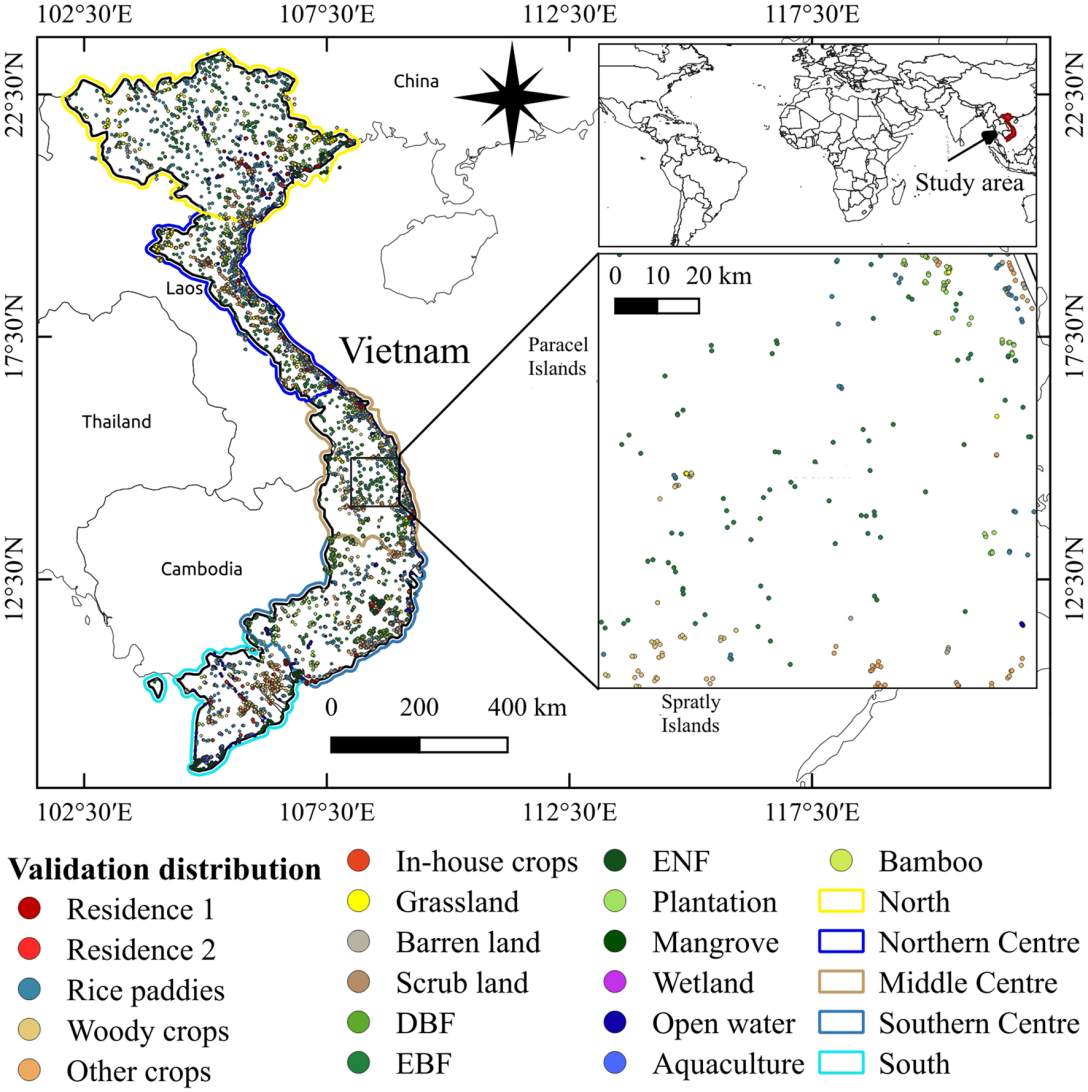
Location of mainland Vietnam in the world: major division zones (**bold lines**), distribution of validation data points across the country. These points are independent from the training data. This figure is generated using QGIS 3.18.0-Zurich (https://qgis.org/en/site/) while the country boundary is extracted from the GADM (https://gadm.org/about.html).

The study area is mainland Vietnam with a population of 97 million people (2018; Fig. 11). The country covers an area of over 300,000 km^2^ including the Red River Delta, and the Mekong River Delta which is the third-largest delta in the world. The topography of Vietnam is diverse (up to 3300 m altitude) with over 75% of the total area being hills and mountains. These areas are covered by mainly tropical rainforests. Climate is changeable but dominated by a tropical monsoon type with mean annual humidity of 84%, mean annual rainfall from 1200 to 3000 mm, and mean annual temperature from 5 to 37 °C^54^. The complex patterns of climate and topography create the rich biodiversity and landscape heterogeneity of Vietnam’s LULC. Nonetheless, there are the identifying characteristics of LULC in different regions. While the southern region is principally occupied with rice, orchards and aquaculture lands, the northern region is primarily covered by forests and plantations, except for the Red River Delta. Dominant LULC types in the northern centre are evergreen broadleaf forests and annual croplands whereas woody crops, deciduous broadleaf forests, and evergreen needle-leaf forests dominate the southern centre. In this study, to reduce the complexity of the landscape information, we divided the whole country into five main regions and separately classified for each region. These regions are presented in Fig. 11.

### Land cover classification system

Defining a standard land cover classification system (LCCS) is a crucial step in the practical land cover assessment. It should be delineated precisely depending on the objectives of users and the availability of mapping resources. Most LULC maps employ the theory and framework of the International Geosphere-Biosphere Programme (IGBP)^55^, the Land Cover Classification System (LCCS)^37^, and the Coastal Change Analysis Program (C-CAP) Land Cover Classifications^56^. Meanwhile, the most updated LCCS of previous LULC products, covering entire Vietnam, includes 18 land cover categories^57^. However, some categories are inappropriate for Vietnam’s LULC. For example, snow and ice do not exist, while one cropland category does not represent the diverse croplands in Vietnam. Although detailed classifications of high and low developed built-up areas play a fundamental role in urban planning and management for the rapid urbanization of Vietnam, they are not in the previous LULC products. In this study, therefore, a new LULC classification system or topology was developed by remaining the appropriate categories of the Food and Agriculture Organization (FAO) LCCS and adding new proper categories based on the local biophysical environment and end-users’ recommendations in Vietnam. First, we classified a 10-category system of primary dominant land use/cover (PDLC/Level-1). The PDLC was then separated into more detailed land types to generate an 18-category system of secondary dominant land use/cover (SDLC/Level-2). We found that this system is appropriate for practically mapping and applications. The categories and descriptions of the system are presented in Supplementary Table S1.

### Remote sensing data

Multi-sensor remote sensing data were used in this study. The data were pre-processed and derived from the GEE. The data were re-projected to Universal Transverse Mercator (UTM) projection (Zone 47-49 N and WGS-84 datum) and then resembled into a 30-m spatial resolution using a bicubic interpolation method^58^. The Geospatial Data Abstraction Library (GDAL), the Geographic Resources Analysis Support System, and Python were utilized for these processing tasks. Specifically, the data included the United States Geological Survey (USGS) Landsat TM, ETM+, and OLI Surface Reflectance Tier 1 with a 30-m spatial resolution, Sentinel MSI Level-2A and SAR GRD with a 10-m spatial resolution. The Landsat and Sentinel MSI have been atmospherically corrected while each scene of Sentinel SAR GRD was preprocessed using Sentinel-1 Toolbox for thermal noise removal, radiometric calibration, terrain correction using the Shuttle Radar Topography Mission (SRTM)^59^, and then converting to decibels^60^. Landsat ETM + images, after the Scan Line Corrector failure in 2003, were removed from this study since the failure may result in inconsistently time-series comparison. Over 99% of the datasets from the GEE archive are reported to have high geometric accuracy with the error being less than half a pixel^27^. Otherwise, the images were eliminated from our image collection to reduce the obvious bias of further analysis.

For reliable and consistent time-series analysis, further processing is integral. For the optical data, to reduce illumination impacts from elevation, aspect and slope, the topographic correction was performed using the Modified Sun-Canopy-Sensor Topographic Correction algorithm^61^. While the Landsat Ecosystem Disturbance Adaptive Processing System (LEDAPS) ^62^ was applied to perform atmospheric correction for Landsat TM and ETM+, the Land Surface Reflectance Code (LaSRC)^63^ was adopted for Landsat OLI. All Landsat images were masked and removed clouds, cloud shadows and saturation pixels utilizing the Function of Mask (CFMASK)^64^. Sen2Cor was adopted to correct atmospheric issues and mask clouds for Sentinel MSI^65^. Finally, because of the different solar and view angles of Landsat OLI and Sentinel MSI, normalizing the bidirectional reflectance distribution function (BRDF) was applied for the data. Although numerous approaches have developed for BRDF correction, the recent technique generated by Roy et al.^66^ is frequently utilized due to its reliability and effective implementation^67^. This method, therefore, was employed for the BRDF correction of all selected optical images in this study. For the Sentinel SAR GRD data, a further process was speckle filtering. The filtering was done using Lee filter, which is superior due to its capacity of maintaining point targets, edge, linear spaces and texture information^68^.

To increase the availability of cloud-free composite data, the harmonization of different Landsat satellite sensor images, and the Landsat OLI with Sentinel MSI into a congruent time-series was desirable for a cloudy region such as Vietnam. The harmonization allows to accurately compare across all years and to measure the spectral similarity and spectral distance between different years. The measurement of spectral similarity and spectral distance was applied for an automatic training migration model, which was described in the following sections. A linear transformation with band-respective coefficients was applied for the harmonization of Landsat TM and ETM + spectral feature to OLI spectral feature^69^. In the meantime, the harmonized Landsat OLI and Sentinel MSI images were processed by employing a method developed by Claverie et al.^70^. The band-respective coefficients with slope and intercept image constants are presented in Table 3. After that, we generated composites of seven bands including blue, green, red, nir, swir 1, swir 2 and thermal bands for two seasons, the dry season from April to September and the wet season from October to March of the following year. These composites were adopted to measure a variety of covariates, which is represented in the following paragraphs. In addition, seasonal composites of VV and VH polarization in ascending and descending orbits of Sentinel SAR GRD were handled in this research.

**Table 3:** Band-respective coefficients are defined with slope and intercept image constants and used for the harmonized Landsat OLI and Sentinel MSI images.

Band-respective		Blue	Green	Red	NIR	SWIR 1	SWIR 2
Harmonizing Landsat TM, ETM + and OLI	Intercept	0.0003	0.0088	0.0061	0.0412	0.0254	0.0172
	Slope	0.8474	0.8483	0.9047	0.8462	0.8937	0.9071
Harmonizing Landsat OLI and Sentinel MSI	Intercept	- 0.0107	0.0026	-0.0015	0.0033	0.0065	0.0046
	Slope	1.0946	1.0043	1.0524	0.8954	1.0049	1.0002

## Satellite-based covariate calculation

In this section, a series of covariates was calculated from the band composites. For the optical data, we calculated the medoid^71^ and the standard deviation for the six bands (Table 3). Following the successful application of numerous features extracted from original satellite image bands, this study also added the medoid of the 20th and 80th percentile^57^ of the six bands into the seasonal composites to detect the seasonal changes in the biophysical environment.

The ratios between spectral bands were calculated; they are blue/green, red/blue, red/green, red/nir and nir/(red*swir 1). Besides, a great number of spectral indices were also measured from Landsat TM, ETM + and OLI, and Sentinel MSI images (Eqs. (1–17); Table 4). In addition, we calculated the seasonal mean of VH, VV, and the normalized difference between VH and VV polarizations from the Sentinel SAR GRD images. Finally, we generated seasonal composite collections of covariates.

**Table 4:** Spectral indices derived from Landsat TM, ETM + and OLI, and Sentinel MSI satellite images to enhance the accurate performance of Vietnam-wide annual LULC mapping from 1990 to 2020.

Name	Equation	No.	Ref
Atmospherically Resistant Vegetation Index	$ARVI = \frac{Bnir - 2Bred + Bblue}{{Bnir + 2Bred + Bblue}}$	(1)	^72^
Difference Vegetation Index	$DVI = Bnir - Bred$	(2)	^99^
Enhanced Built-Up and Bareness Index	$EBBI = \frac{Bswir - Bnir}{{10\sqrt {Bswir + Bnir} }}$	(3)	^100^
Enhanced Vegetation Index	$EVI = 2.5\frac{Bnir - Bred}{{Bnir + 6Bred - 7.5Bblue + 1}}$	(4)	^101^
Green Chlorophyll Index	$GCI = \frac{Bnir}{{Bgreen}} - 1$	(5)	^102^
Mangrove Vegetation Index	$MVI = \frac{Bnir - Bgreen}{{Bswir - Bgreen}}$	(6)	^80^
Normalized Burn Ratio	$NBR = \frac{Bnir - Bswir}{{Bnir + Bswir}}$	(7)	^81^
Normalized Different Bareness Index	$NDBaI = \frac{Bswir - Btir}{{Bswir + Btir}}$	(8)	^79^
Normalized Difference Built-Up Index	$NDBI = \frac{Bswir - Bnir}{{Bswir + Bnir}}$	(9)	^91^
Normalised Difference Pond Index	$NDPI = \frac{Bgreen - Bswir}{{Bgreen + Bswir}}$	(10)	^92^
Normalized Difference Turbidity Index	$NDTI = \frac{Bred - Bgreen}{{Bred + Bgreen}}$	(11)	^92^
Normalized Difference Vegetation Index	$NDVI = \frac{Bnir - Bred}{{Bnir + Bred}}$	(12)	^93^
Normalized Difference Water Index	$NDWI = \frac{Bgreen - Bnir}{{Bgreen + Bnir}}$	(13)	^94^
Soil Adjusted Vegetation Index	$SAVI = 1.5\frac{Bnir - Bred}{{Bnir + Bred + 0.5}}$	(14)	^95^
Structure Insensitive Pigment Index	$SIPI = \frac{Bnir - Bblue}{{Bnir - Bred}}$	(15)	^96^
Urban Index	$UI = \frac{Bswir - Bnir}{{Bswir + Bnir}}$	(16)	^97^
Water Ratio Index	$WRI = \frac{Bgreen + Bred + Bnir}{{Bblue}}$	(17)	^98^

### Ancillary data sets

Extensive research has shown that ancillary information can improve the accurate performance of LULC classification^20,82,83^. In this study, we first added terrain indices including slope, aspect and elevation. These indices were computed from ALOS Global Digital Surface Model or “ALOS World 3D-30 m (AW2D30)”^84^. Also, distance to rivers, coastlines, transport systems and buildings, and soil types were included in the covariate collections. The buildings and transport systems were generated from the OpenStreetMap, while soil types and river networks were extracted from the OpenDevelopmentMekong^86^.

### Reference data

Reference data of 18 LULC categories (Supplementary Table S1) was created from field surveys, provincial LULC statistics, and visual interpretations. We conducted nationwide comprehensive surveys in 2015, 2016, 2018, 2019, and 2020 to collect 3078, 2659, 10,550, 41,986 and 32,853 reference samples, respectively. Along with these ground-observed data, previous outdated LULC maps^19,20,87^, provincial LULC statistics and high-resolution satellite images available in the Google Earth were also considered. Herein, we generated approximately 9360 polygons of single homogeneous LULC types (Fig. 11) throughout the country for each year from 2015 to 2020. From these polygons, we extracted up to 120,000 reference pixels (points) for each of the years. Meanwhile, due to the non-availability of ground-truth data, reference data from 1990 to 2014 were collected using provincial LULC statistics, the natural-color images of Landsat TM, ETM + and OLI, Sentinel MSI, and high-resolution satellite images available in the Google Earth. For each year, we randomly extracted 1050 points per LULC category for validating the classification models and the others were used for training the classification model.

The reference data of the year 2020 were utilized not only for creating and validating the LULC map of the year 2020 but also for implementing the automatic training migration model from this reference year to any target years. The migration model is described in more detail in the following section.

### Automatic training migration model

Training data is tremendously essential in mapping LULC; however, collecting sufficiently accurate training samples is challenging, especially for large-scale areas, long-term history analyses, and data-scarce environments such as Vietnam^73^. If training data are not collected consistently, it can result in misclassification or low accuracies^74^. Thus, it is paramount to propose a practical approach for training data collections.

In this study⁠, we utilized an automatic model to migrate from the reference data of a reference year to target years. The method had three essential steps. First, we created a set of training data from a reference year (2020). Then, for each pixel, we computed its surface reflectance values from its corresponding Landsat TM, ETM +, and OLI images of the reference year and target years. The surface reflectance values of six bands (Table 3) were utilized for the measurement of Euclidean distance (ED)^75^ and spectral angle distance (SAD)^76^. Finally, with the ED (Eq. 18) and SAD (Eq. 19), we distinguished changed pixels and unchanged pixels by running a trial and error model to determine thresholds. Although the thresholds can be estimated by analysts, the experimented thresholds of ED and SAD in this study was 0.05 and 0.95, respectively. These thresholds were successfully applied to migrate the training data of the year 2020 to target years. The unchanged pixels were preserved and utilized as training data for the target years.

18 $$ED = \sqrt {\mathop \sum \limits_{i = 1}^{N} \left( {X_{i} - Y_{i} } \right)^{2} }$$

19 $$SAD = arccos\left( {\frac{{\mathop \sum \nolimits_{i = 1}^{N} X_{i} Y_{i} }}{{\sqrt {\mathop \sum \nolimits_{i = 1}^{N} \left( {X_{i} } \right)^{2} \mathop \sum \nolimits_{i = 1}^{N} \left( {Y_{i} } \right)^{2} } }}} \right)$$

where *X* is spectral signature vector of an image pixel in the reference year; *Y* is spectral signature vector of an image pixel in the target year; *N* is the number of image bands (*N* = 6).

### Machine learning modelling

After completing the data preprocessing task, we generated covariate collections, including (1) optical-image-based covariates in dry seasons and (2) in wet seasons, and (3) SAR-based covariates in dry seasons and (4) in wet seasons. The ancillary information was also added to these covariate collections. It is worth noting that some of these covariates or features may not significantly contribute to the enhancement of classification performance while overabundant features can affect the performance speed or run out of computing capacity of the classification model. Hence, random forest algorithm^77^ was employed to estimate important features. We removed some less important features and kept essential features which were represented in Supplementary Table S2.

For classification, we applied a random forest algorithm for several reasons. First, it has previously been observed that the random forest algorithm can handle principal drawbacks that a single-tree-based method may face such as an over-fitting and non-optimal solution^78^. Also, the random forest shows the outperformance of its rivals such as fuzzy adaptive resonance theory-supervised predictive mapping (Fuzzy ARTMAP), support vector machine (SVM), artificial neural network (ANN), Mahalanobis distance (MD), and spectral angle mapper (SAM)^39^.

A new random-forest-based approach was developed in this study. Unlike the common use of single-time classification, for each pixel, we independently estimated prior probability values belonging to each of the specified land covers for each of the covariate collections. These prior probability values were then joined to create a set of posterior probability values. The largest value of the posterior probabilities corresponding to a specific land cover was utilized to label the predicted land cover. However, the predicted prior probability of a pixel, for example, *p*(*C*_*k*_), might reach almost zero or zero because of ‘*No data*’ of that pixel at that covariate collection. If this occurs, the posterior probability of that pixel will be nearly zero or zero. That is, although the prior probability of most other collections equals 100% voting for a specified land cover, the probability product of this pixel might be almost zero, causing misclassification. Hence, the prior probability of a pixel corresponding to a particular land cover must not be extremely tiny. To this end, Eq. (20) was developed to adjust the prior probability values while posterior probability values were calculated as Eq. (21).

20 $$p^{,} \left( {C_{k} } \right) = c*p\left( {C_{k} } \right) + \frac{1 - c}{N}$$

21 $$p_{c} \left( {C_{k} } \right) = \mathop \prod \limits_{i = 1}^{I} p_{i}^{,} \left( {C_{k} } \right)$$

where *p*’(*C*_*k*_) is the adjustment of prior probability value of a land cover *C*_*k*_; *c* is a constant value (*c* = 0.7) which was evaluated by trial and error experiments in this study; *N* is the number of land cover categories (*N* = 18); *p*_*c*_(*C*_*k*_) is the posterior probability value of category *C*_*k*_; and *I* is the number of covariate collections (*I* = 4).

The random-forest-based model was performed using Scikit-Learn 0.22 and Python 3.8.5. Since the input data of multi-sensor image bands and covariates dramatically varied over the 30-year period, we could not optimize all the parameters of the random forest algorithm. The number of trees (n_estimators) in the forest and the size of the random subsets of features (max_features), however, are highly recommended to be adjusted^77^. Using RandomizedSearchCV in the Scikit-Learn, we found that n_estimators = 200 (trees) and max_features = 8 were optimal in this work. The other parameters were set as the default values.

### Accuracy assessment

Following the wide-ranging recommendations of instruction manuals^88,89^, a statistic-based testing data set (Reference data section) was independently generated to estimate the accuracy of final LULC products. We utilized a stratified sampling (1050 points/LULC category) method and a confusion matrix to assess Vietnam-wide annual LULC products from 1990 to 2020. The matrix produced profound accuracy metrics, namely overall accuracy (OA), user accuracy (UA), standard error (SE), and kappa coefficient (KC). The uncertainty of accuracy was measured with a 95% confidence interval. These metrics are fully described in Tables 1 and 2.

### Change analysis

The analysis of changes in LULC is to measure the differences including spatio-temporal dynamic patterns, the magnitude, and rate of variations observed over the study period. First, we estimated the diversity of LULC within each 30-m pixel width by counting the number of times that LULC changes over 30 years (Fig. 1c). We then estimated the area of each LULC within a five-year interval from 1990 to 2020 to observe the trend of LULC change (Fig. 5). We also computed the percentage of net change (Eq. 22) and then rescaled the percentage to a rank between 0 and 100% to monitor the most dynamic LULC (Fig. 6). Finally, we employed a Sankey diagram to emphasize the major transfers of LULC^90^.

22 $$p = \left( {\frac{{A_{t2} - A_{t1} }}{{A_{t1} *\left( {t_{2} - t_{1} } \right)}}} \right)*100$$

where *p* is the percentage of net change; and $A_{t1}$ and $A_{t2}$ (km^2^) are the area of the LULC type in the observation years $t_{1}$ and $t_{2} ,$ respectively (*t*_1_ < *t*_2_).

## Supplementary Information

Supplementary Information.
