# Supplementary material for: First comprehensive quantification of annual land use/cover from 1990 to 2020 across mainland Vietnam
Source: Sci Rep. 2021 May 11;11:9979. doi: 10.1038/s41598-021-89034-5 (PMC8113344; doi:10.1038/s41598-021-89034-5)
Supplement: Supplementary file 1 — Supplementary Information. [file 41598_2021_89034_MOESM1_ESM.pdf]

First comprehensive quantification of annual land use/cover from 1990 to 2020 across mainland Vietnam.

Duong Cao Phan[1,2,*], Ta Hoang Trung[3], Van Thinh Truong[4], Taiga Sasagawa[1], Thuy Phuong Thi Vu[5], Dieu Tien Bui[6], Masato Hayashi[7], Takeo Tadono[7], and Kenlo Nishida Nasahara[8].

[1]Graduate School of Science and Technology, University of Tsukuba, Tennoudai 1-1-1, Tsukuba, Ibaraki 305-8572, Japan; s1936042@s.tsukuba.ac.jp; sasagawataiga.ryuiki@gmail.com.

[2]Hydraulic Construction Institute—Vietnam Academy for Water Resources, No. 3, Alley 95, Chua Boc Street, Dong Da district, Hanoi 116765, Vietnam; pcduong8088@gmail.com.

[3]Department of Survey, Mapping and Geographic Information, Ministry of Natural Resources and Environment, 2 Dang Thuy Tram street, Hanoi 100000, Vietnam; tahoangtrung@gmail.com.

[4]VNU Center for Development in Hoa Lac, Vietnam National University, Hanoi, Thach Hoa Commune, Thach That District, Hanoi 155500, Vietnam; s2036038@s.tsukuba.ac.jp.

[5]Forest Inventory and Planning Institute (FIPI), Ministry of Agriculture and Rural Development (MARD), Vinh Quynh, Thanh Tri, Hanoi 100000, Vietnam; s1926065@s.tsukuba.ac.jp.

[6]GIS group, Department of Business and IT, University of South-Eastern Norway, Gullbringvegen 36, N-3800 Bø i Telemark, Norway; dieu.t.bui@usn.no

[7]Earth Observation Research Center, Japan Aerospace Exploration Agency (JAXA), 2-1-1 Sengen, Tsukuba, Ibaraki 305-8505, Japan.

[8]Faculty of Life and Environmental Sciences, University of Tsukuba, Tennoudai 1-1-1, Tsukuba, Ibaraki 305-8572, Japan; nasahara.kenlo.gw@u.tsukuba.ac.jp.
[*]pcduong8088@gmail.com

Primary dominant land cover	Code	Secondary dominant land cover	Description
Residential land	R1	High developed areas	Land covered by buildings and other man-made structures > 50 % of constructed cover and < 20 % vegetation.
	R2	Low developed areas	Land covered by buildings and other man-made structures < 50 % of constructed cover and < 20 % vegetation.
Rice paddies	RP	Rice paddies	Rice fields have a area of greater than 30 x 30 m.
Cropland	WC	Woody crops	Perennial woody crops such coffee, tea, woody orchards, and so on
	IC	In-house crops	Crops are planted in agricultural plastic houses, which have an area go larger than 30 m by 30 m.
	OC	Other croplands	Lands covered with temporary crops followed by harvest and a bare soil period (e.g., single and multiple cropping systems). It is noted that perennial woody crops will be classified as the woody crops or the appropriate forest or shrub land cover type.
Grassland	GL	Grassland/ Herbaceous vegetation	> 80 % of herbaceous land; not intensive management but can be utilized for grazing. Tree and shrub cover is less than 10 %
Barren land	BL	Barren land	Lands with exposed soil, sand, rocks, or snow and never have more than 10% vegetated cover during any time of the year.
Scrub land	SL	Scrub/Shrub	Areas are dominated by shrubs less than 5 % of 5 meter tall. Shrub canopy is typically greater than 10 % of total vegetation. This category includes tree shrubs, young trees in an early successional stage, or trees stunted from environmental conditions.
Forest land	DBF	Deciduous broadleaf forest	Lands dominated by woody vegetation with a percent cover > 60 % and height exceeding 5 m. Consisting of broadleaf tree communities with an annual cycle of leaf-on and leaf-off periods
	EBF	Evergreen broadleaf forest	Lands dominated by broadleaf woody vegetation with a percent cover > 60 % and height exceeding 5 m. Almost all trees remain green year round. Canopy is never without green foliage.
	ENF	Evergreen needle-leaf forest	Lands dominated by needle-leaf woody vegetation with a percent cover > 60 % and height exceeding 5 m. Almost all trees remain green all year. Canopy is never without green foliage.
	PL	Plantation land	Lands dominated by plantation trees with a percent cover > 60 % and height exceeding 5 m.
	BA	Bamboo area	Lands dominated by bamboo with a percent cover > 60 %.
Wetland	IW	Inland wetland	Include tidal and non-tidal wetlands dominated by vegetation. Note that mangrove is classified as the mangrove forests
	MF	Mangrove	Lands dominated by mangrove trees with a percent cover > 60 % and height exceeding 5 m.
Open water	OW	Open water	Oceans, seas, lakes, reservoirs, and rivers which can be either fresh or saltwater bodies.
Aquaculture	AC	Aquaculture	Contains areas dominated by aquatic organism and plant farming, including artificial ponds and temporarily flooded areas.

Supplementary Table S1. Land use/cover classification system.

Data source	Order	Covariates	Resolution (m)	
			1990 – 2014	2015 – 2020
Landsat TM, ETM+ and OLI, and Sentinel MSI	1	blue	30	10
	2	green	30	10
	3	red	30	10
	4	nir	30	10
	5	swir1	30	10
	6	swir2	30	10
	7	thermal	30	10
	8	p20_green	30	10
	9	p20_nir	30	10
	10	p20_blue	30	10
	11	p20_red	30	10
	12	p20_swir1	30	10
	13	p20_swir2	30	10
	14	p80_green	30	10
	15	p80_nir	30	10
	16	p80_blue	30	10
	17	p80_red	30	10
	18	p80_swir1	30	10
	19	p80_swir2	30	10
	20	blue_stdDev	30	10
	21	red_stdDev	30	10
	22	green_stdDev	30	10
	23	nir_stdDev	30	10
	24	swir1_stdDev	30	10
	25	swir2_stdDev	30	10
	26	ND_green_swir1_stdDev	30	10
	27	ND_nir_red_stdDev	30	10
	28	ND_nir_swir2_stdDev	30	10
	29	blue/green	30	10
	30	red/blue	30	10
	31	red/green	30	10
	32	red/nir	30	10
	33	nir/(red*swir1)	30	10
	34	ARVI	30	10
	35	DVI	30	10
	36	EBBI	30	10
	37	EVI	30	10
	38	GCI	30	10
	39	MVI	30	10
	40	NBR	30	10
	41	NDBaI	30	10
	42	NDBI	30	10
	43	NDPI	30	10
	44	NDTI	30	10
	45	MDVI	30	10
	46	NDWI	30	10
	47	SAVI	30	10
	48	SIPI	30	10
	49	UI	30	10
	50	WRI	30	10
Sentinel SAR GRD	51	VV	-	10
	52	VH	-	10

	53	ND_VV_VH	-	10
AW2D30	54	elevation	30	10
	55	slope	30	10
	56	aspect	30	10
OpenStreetMap	57	distRoad	30	10
	58	distBuilding	30	10
OpenDevelopmentMekong	59	distCoast	30	10
	60	distRiver	30	10
	61	soilType	30	10

Supplementary Table S2. Bands, indices and ancillary data used to create annual land use/cover maps of Vietnam from 1990 to 2020.
